# Supplementary material for: Temporal dynamics of the developing lung transcriptome in three common inbred strains of laboratory mice reveals multiple stages of postnatal alveolar development
Source: PeerJ. 2016 Aug 9;4:e2318. doi: 10.7717/peerj.2318 (PMC4991849; doi:10.7717/peerj.2318)
Supplement: Data S1 [file peerj-04-2318-s001.docx]

**Mouse Sex Determination Protocol:**

The sex of each embryo and pup less than 21 days of age was determined by PCR with primers against the male specific *Sry* gene (MGI:98660). DNA was extracted from tail fragments collected from every embryo and mouse using the HotSHOT method of preparation as previously described (Truett et al. Biotechniques 2000 29:52-54). Each 20 microliter PCR reaction contained 1 microliter of HotSHOT DNA, 0.5 mM each of SryF and SryR primers, 0.5 mM of each dNTP (Promega, Madison, WI), 1 unit of Phusion taq polymerase (New England Biolabs, Ipswitch, MA) and 1X taq polymerase buffer. PCR reactions were amplified by incubation at 94^0^C for 2 min followed by 40 cycles of incubation at 94^0^C for 30 sec, 64^0^C for 30 sec and 72^0^C for 30 sec then incubation at 72^0^C for 4 min and hold at 4^0^C. PCR products were visualized by electrophoresis through 1.6% agarose gels. The sequences of the *Sry* primers are as follows:

SryF: 5’-CAAGCGCCCCATGAATGCATTTATGG-3’

SryR: 5’-ACACTTTAGCCCTCCGATGAGGCTGA-3’
