# Supplementary material for: Temporal dynamics of the developing lung transcriptome in three common inbred strains of laboratory mice reveals multiple stages of postnatal alveolar development
Source: PeerJ. 2016 Aug 9;4:e2318. doi: 10.7717/peerj.2318 (PMC4991849; doi:10.7717/peerj.2318)
Supplement: Data S7 [file peerj-04-2318-s007.docx]

**Supplemental Data S6. Gene Ontology annotation term enrichment data for the murine Developing Lung Characteristic Subtranscriptome (mDLCS)**

Supplemental data file S6 contains the gene lists and results for annotation enrichment analysis of Gene Ontology terms.

The gene lists include the genes with positive and negative loadings on principal components 1-3 and the combined set of genes from PC1-3 that constitute the mDLCS.

The analysis was performed with version 1.5.1 of Visual Annotation Display (VLAD; <http://proto.informatics.jax.org/prototypes/vlad/>).

Gene Ontology terms and annotations obtained from the Mouse Genome Informatics database (MGI; <http://www.informatics.jax.org>) June 2015.

The column headers for the enrichment results are as follows:

|  | |  |
| --- | --- | --- |
| **TermID** | ID of the ontology term. |  |
| **Term** | The text of the term. |  |
| **Term Min P** | The smallest P-val for any query set for this term. | |
| **Term Max P** | The largest P-val for any query set for this term. | |
| **Term P ratio** | Term Min P divided by Term Max P. | |
| **P** | P value (significance score) for this query set for this ontology term. | |
| **Q** | The False Discovery Rate (FDR) statistic. | |
| **k** | The number of objects in the query set annotated to this term (or a descendant). | |
| **n** | The size of the query set. |  |
| **K** | The number of objects in the database annotated to this term (or a descendant). | |
| **N** | Number of annotated objects in the database. | |
| **k/n** | Percent of query set objects annotated to this term (or a descendant). | |
| **K/N** | Percent of database objects annotated to this term (or a descendant). | |
| **k/K** | Percent of all objects annotated to this term present in the query set. | |
| **n/N** | Percent of database in the query set. | |
| **Qset** | The name of the query set. |  |
| **Symbols** | The symbols query set objects annotated to this term (or a descendant). | |
