# Supplementary figures and images for: Temporal dynamics of the developing lung transcriptome in three common inbred strains of laboratory mice reveals multiple stages of postnatal alveolar development
Source: PeerJ. 2016 Aug 9;4:e2318. doi: 10.7717/peerj.2318 (PMC4991849; doi:10.7717/peerj.2318)

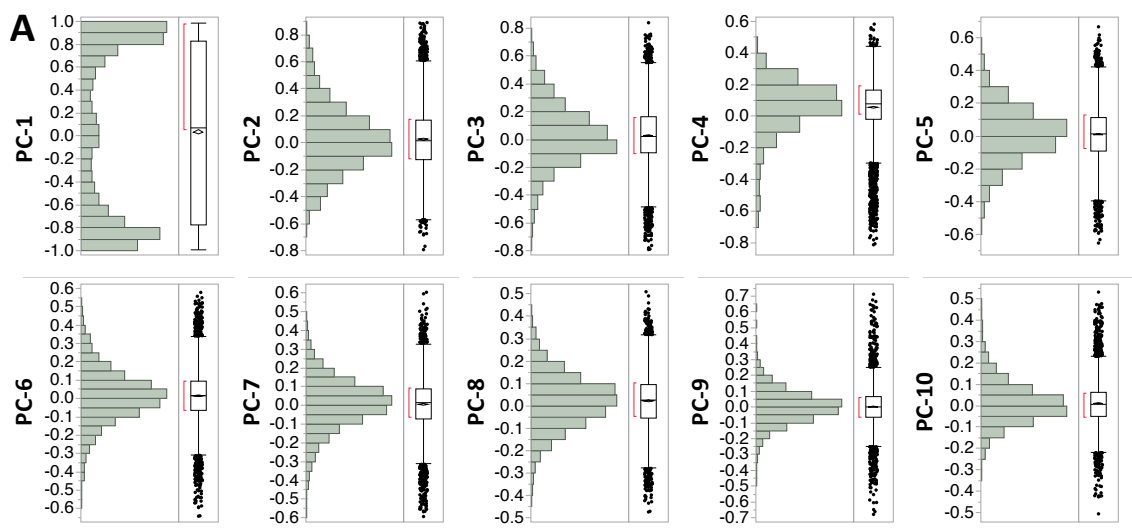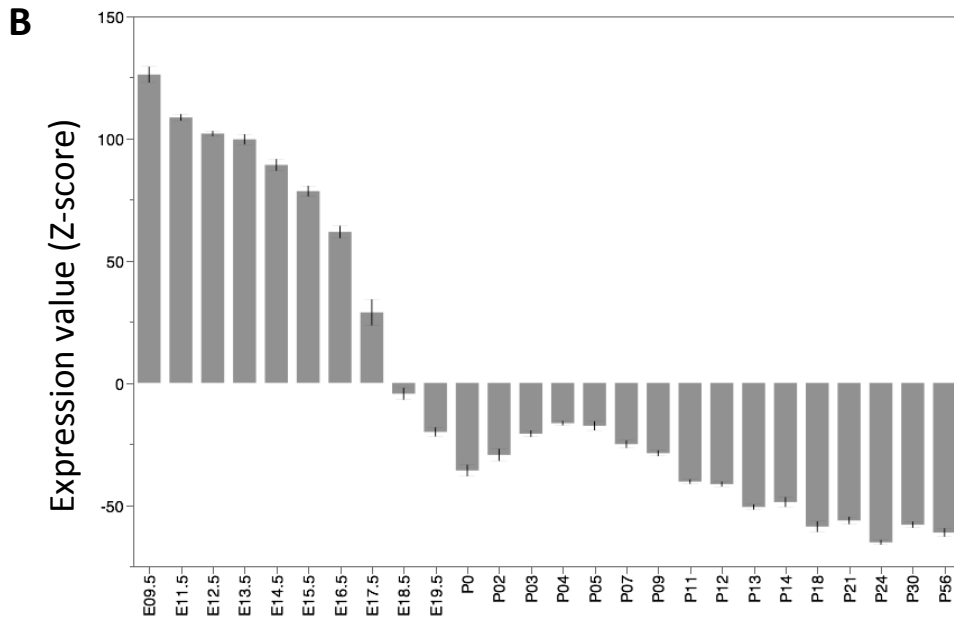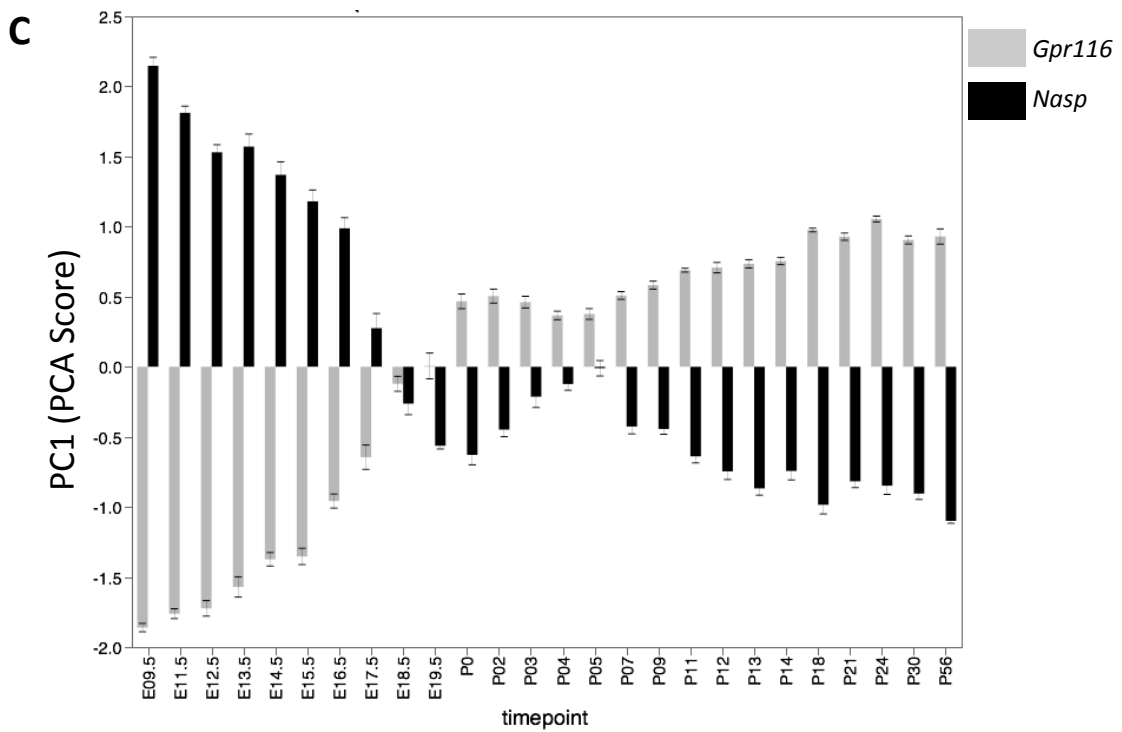

Supplement: Figure S1 — (A) Plots of gene loading values for PC 1–10. (B) Plot of PCA scores for PC1 across lung development time points sampled in this study highlighting a dramatic shift in gene loading values just before birth. (C) Mean expression levels of two representative genes with inverse loading values from PC1. Black bars, expression of Nasp (positive loading value); light grey bars, expression of Gpr116 (negative loading value). Error bars reflect one standard error from the mean. Complete PCA results of gene loading values and PCA scores found in Data S3 and S4, respectively. [file peerj-04-2318-s010.pdf]

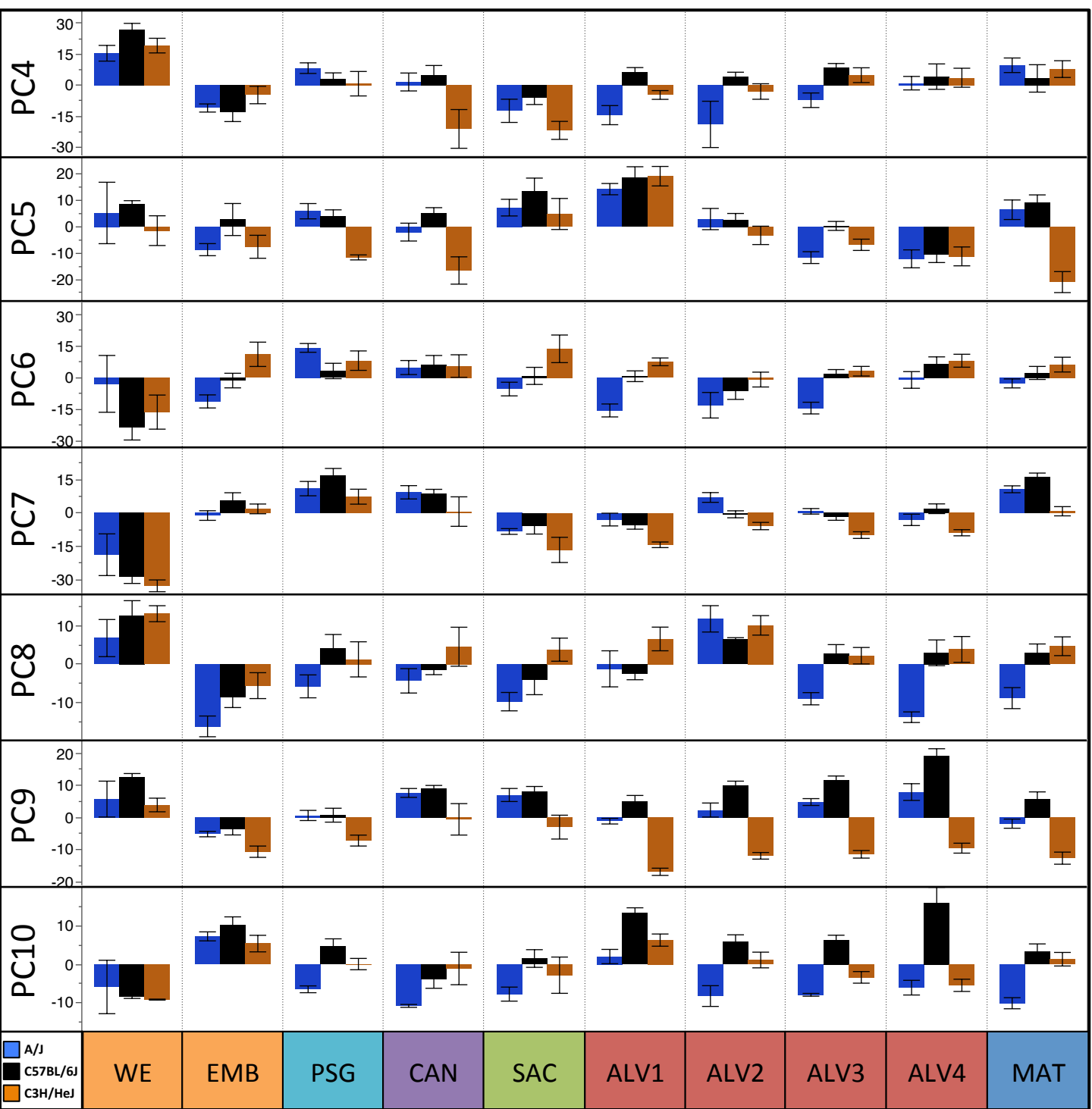

Supplement: Figure S2 [file peerj-04-2318-s011.pdf]

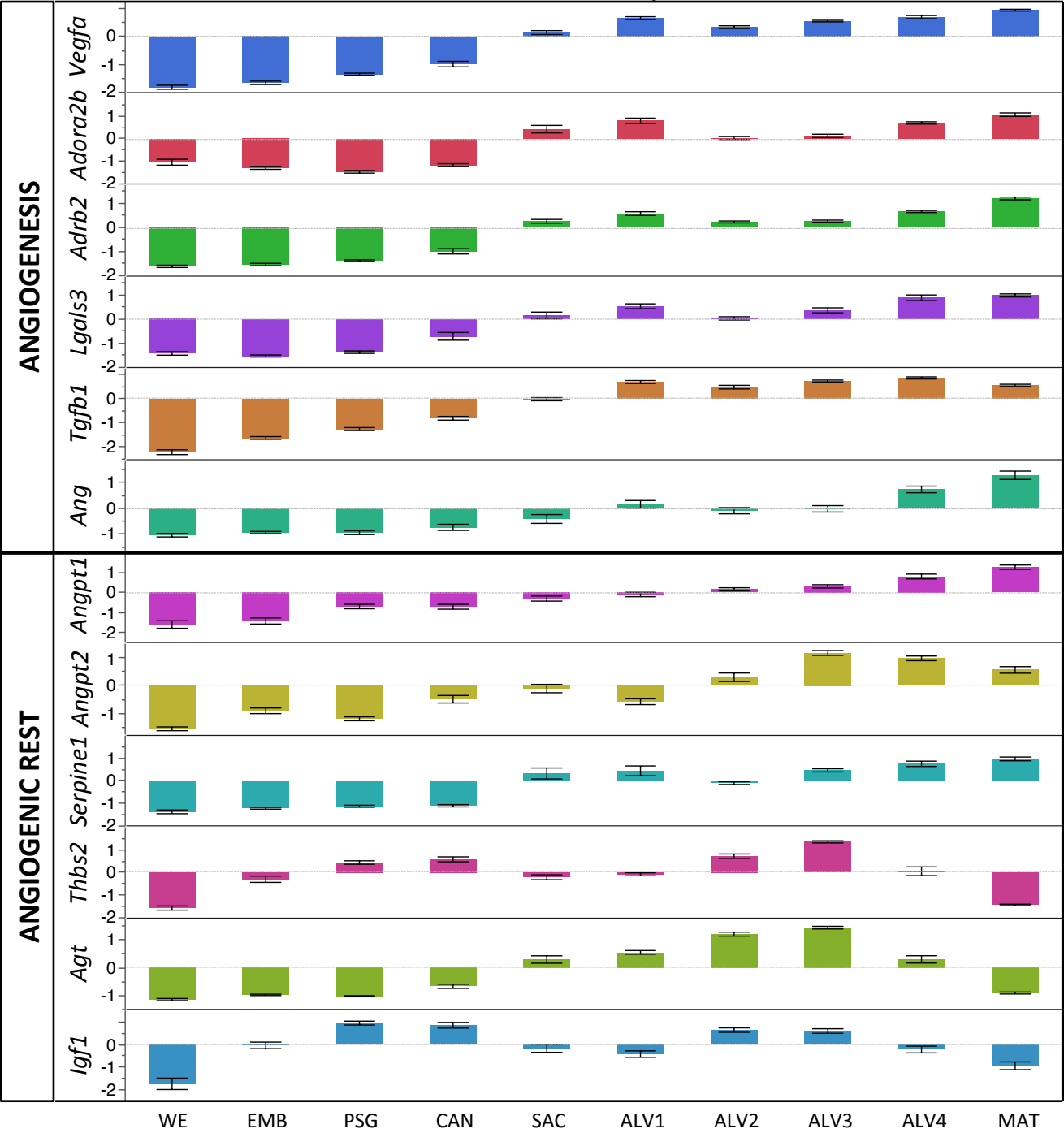

Supplement: Figure S3 — Each error bar is constructed using 1 standard error from the mean. The patterns of expression illustrate the concept of periods of “angiogenic rest” during alveolarization. Potent angiogenic factors (Vegfa, Tgfb1, Ang) and known regulators of pulmonary vascularization (Adora2b, Adrb2, Lgals3) have elevated expression levels at alveolar stages (ALV1 and/or ALV4). Genes associated with the negative regulation of angiogenesis (Thbs2, Agt) and vascular stabilization/maturation factors (Angpt1, Angpt2, Serpine1, Igf1) show an inverse relationship of expression levels. [file peerj-04-2318-s012.pdf]

# LUNG ALVEOLUS DEVELOPMENT

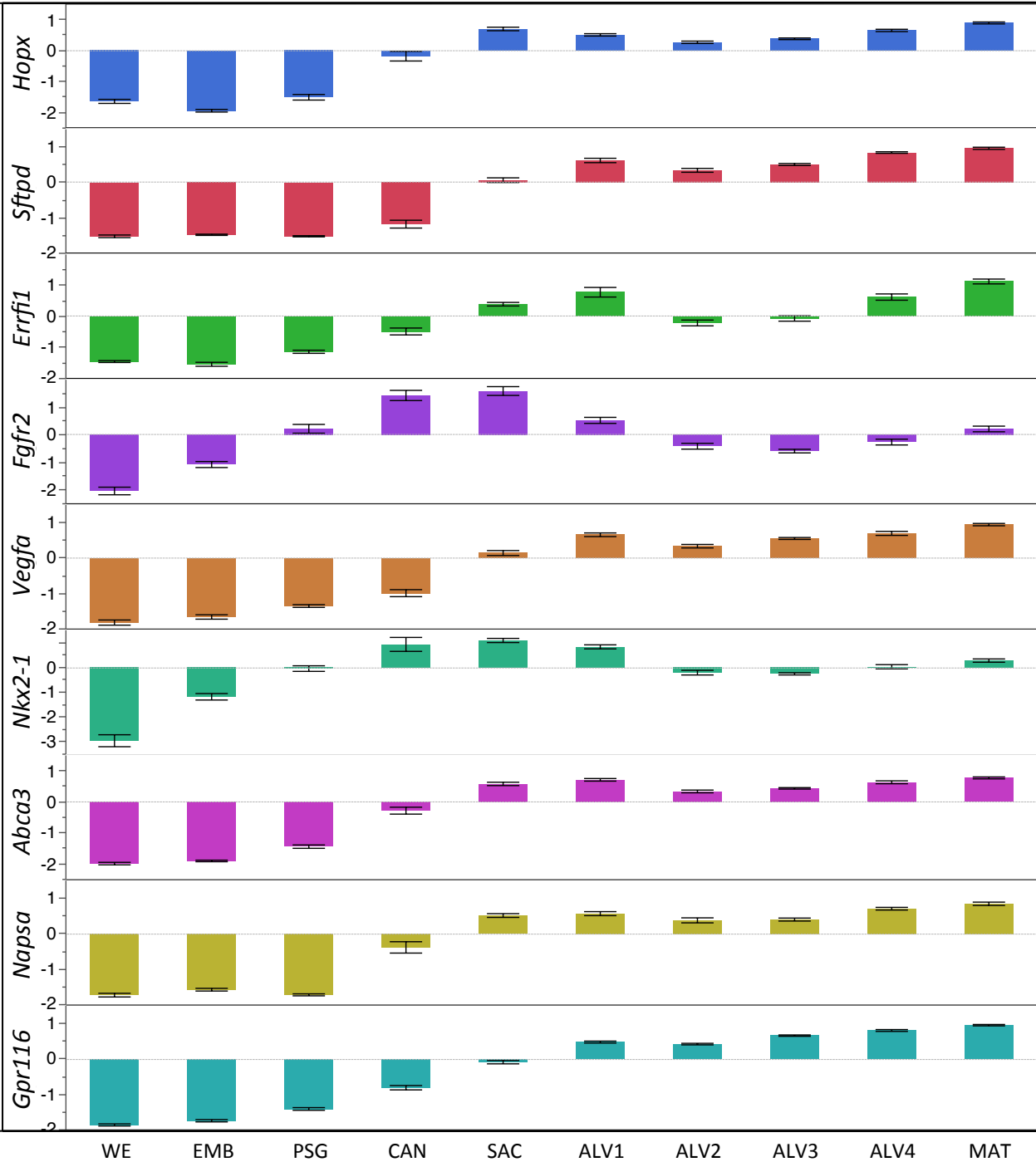

Supplement: Figure S4 — Plot of Z-scores (y-axis) for gene expression levels of selected genes associated with lung alveolus development. Each error bar is constructed using 1 standard error from the mean. Transcription factors (Hopx, Nkx2-1, Errfi1), growth factors (Fgfr2, Vegfa) and genes involved in pulmonary surfactant production (Sftpd, Abca3, Gpr116, Napsa) are differentially expressed between ALV1-ALV2 and/or ALV3-ALV4. [file peerj-04-2318-s013.pdf]

# AXON GUIDANCE / NEUROGENESIS

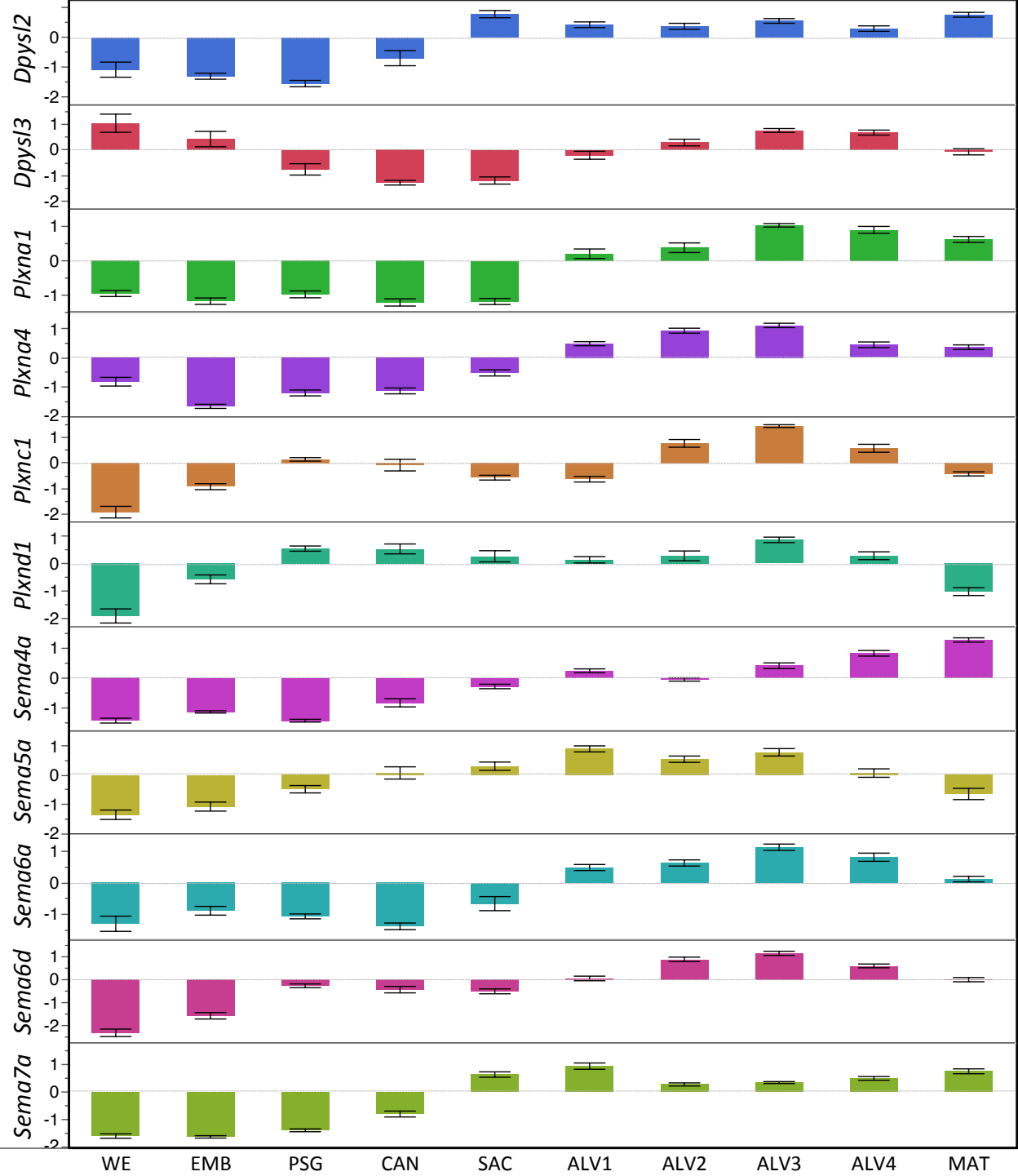

Supplement: Figure S5 — Plot of Z-scores (y-axis) for gene expression levels of selected genes associated with axon guidance and neurogenesis. Each error bar is constructed using 1 standard error from the mean. [file peerj-04-2318-s014.pdf]

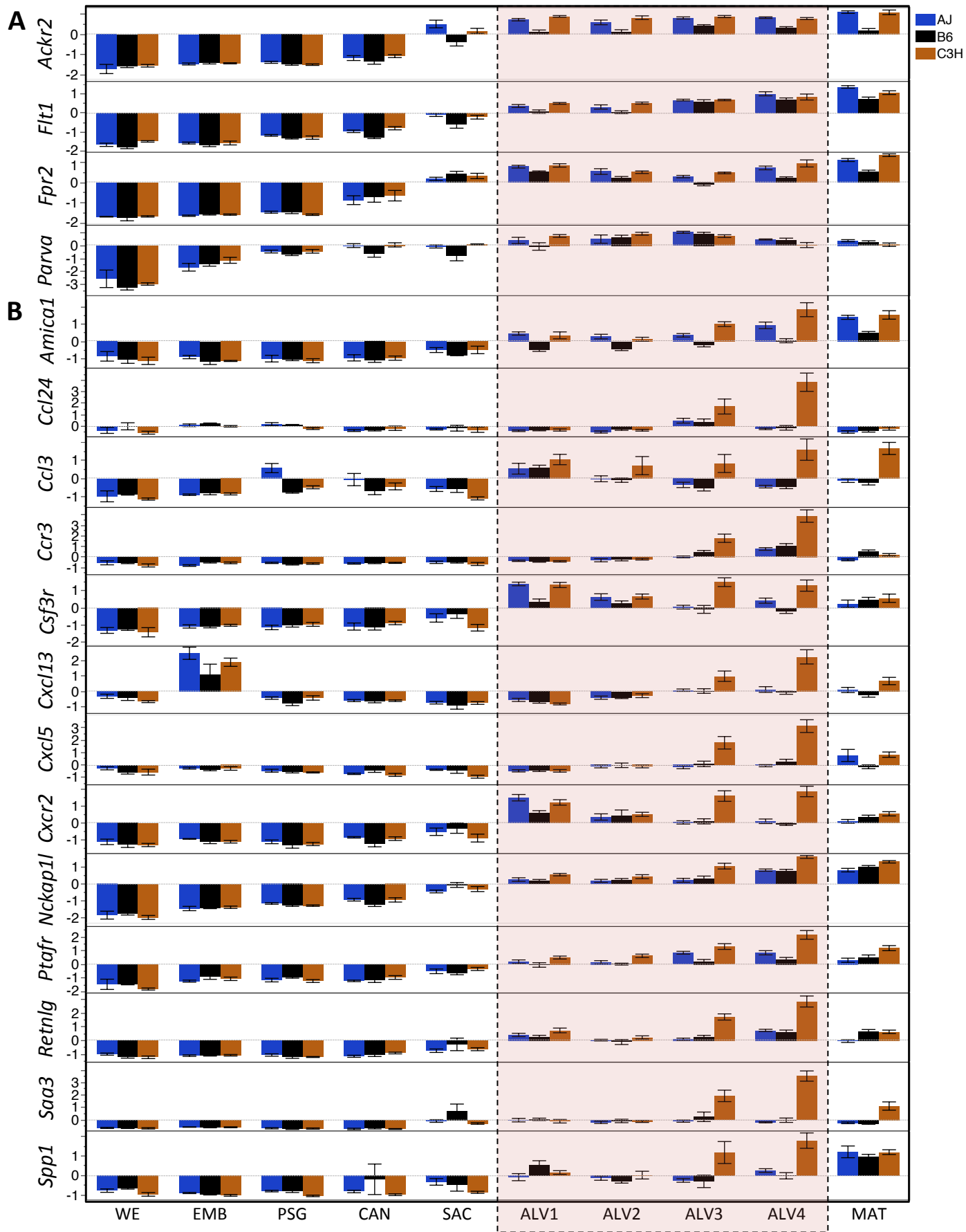

Supplement: Figure S7 — Plot of Z-scores (y-axis) for gene expression levels of genes associated with chemotaxis. Each error bar is constructed using 1 standard error from the mean. (A) Genes associated with chemotaxis with higher expression levels in AJ or C3H relative to B6. (B) Genes associated with immune-related chemotaxis with higher expression levels in C3H relative to AJ or B6. Alveolar stages (ALV1-4) highlighted in red. [file peerj-04-2318-s016.pdf]

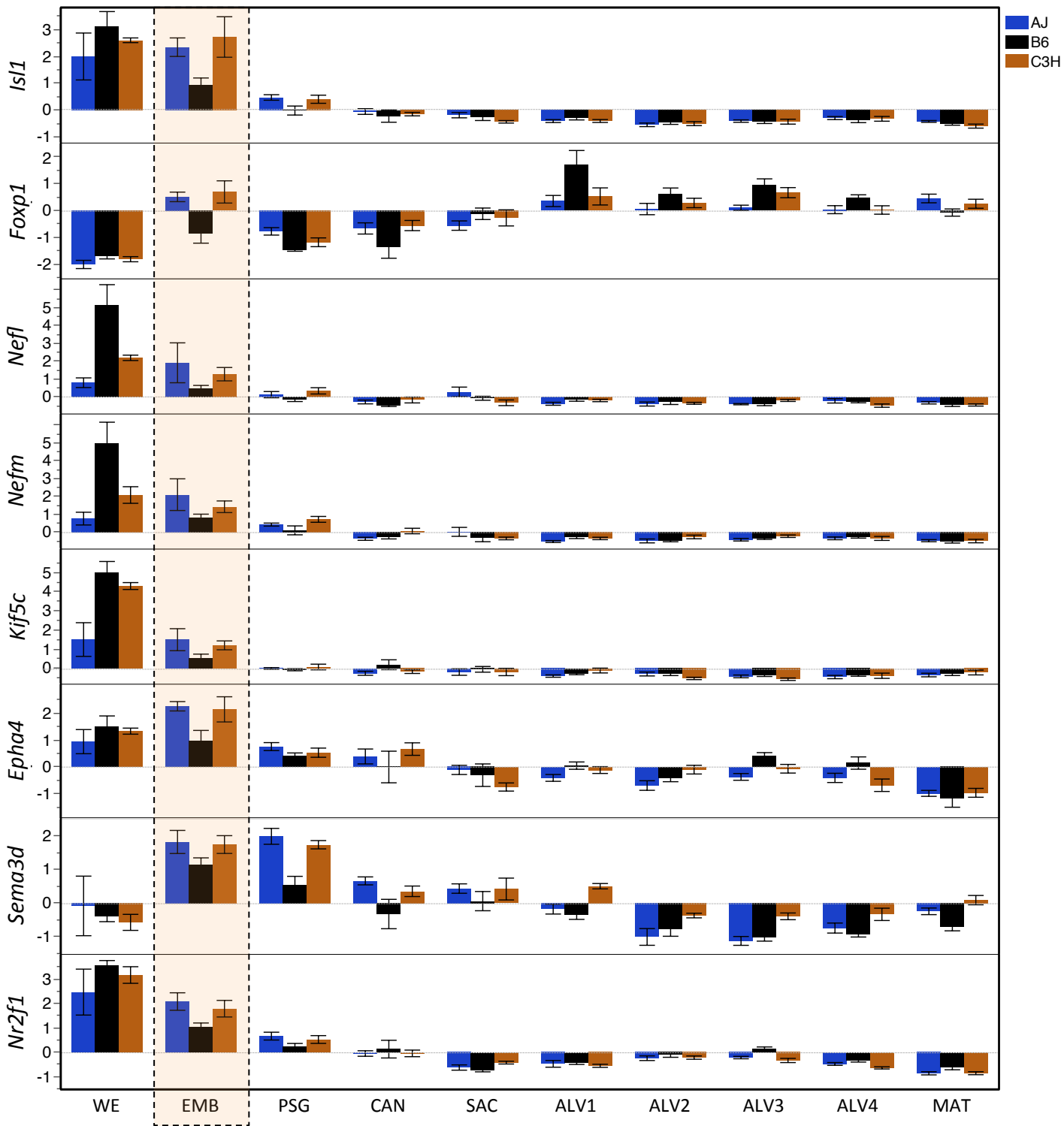

Supplement: Figure S8 — Each error bar is constructed using 1 standard error from the mean. Embryonic stage (EMB) highlighted in orange. [file peerj-04-2318-s017.pdf]

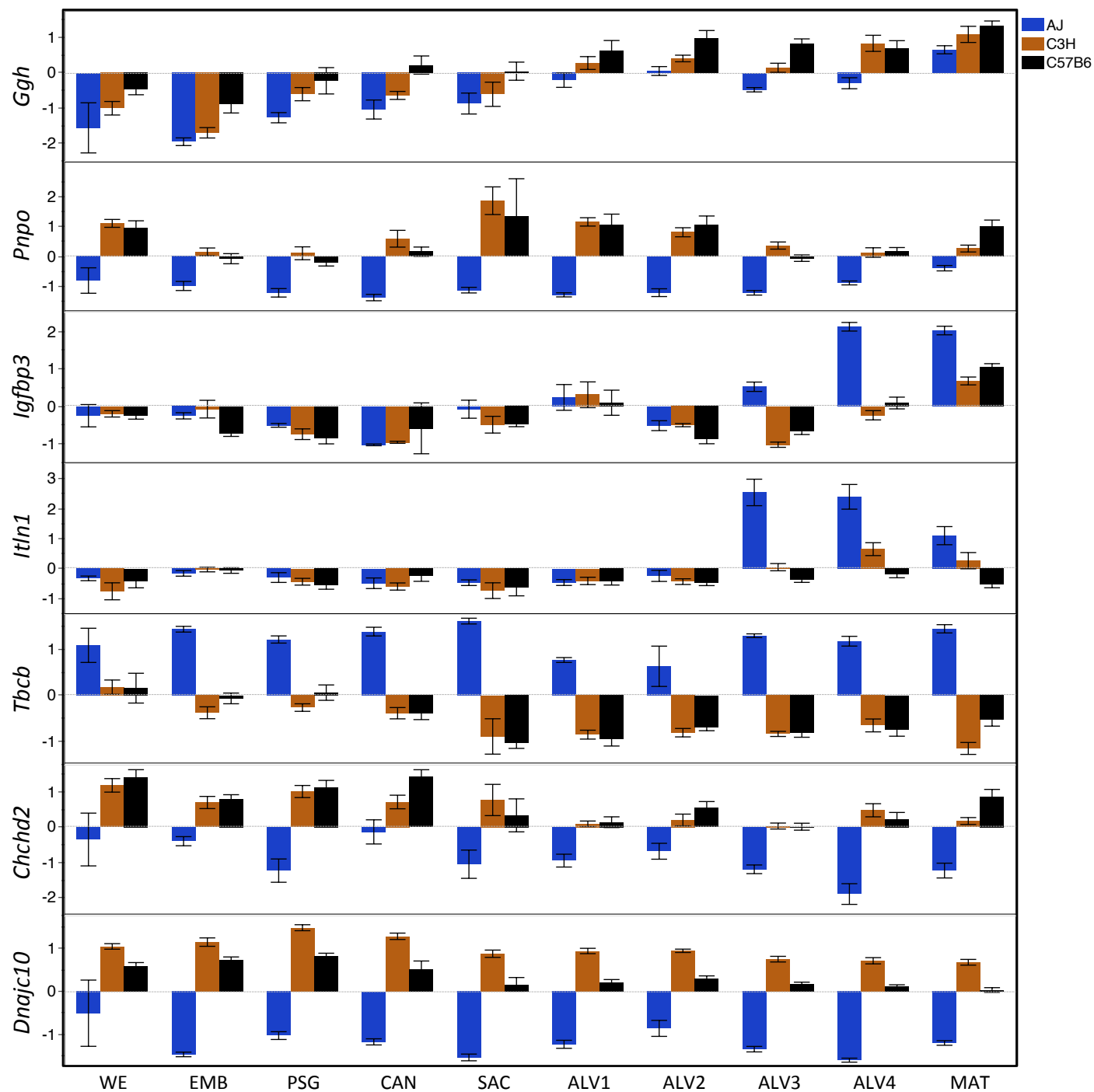

Supplement: Figure S9 — Plot of Z-scores (y-axis) for gene expression levels of genes differentially expressed in AJ relative to C3H or B6. Each error bar is constructed using 1 standard error from the mean. [file peerj-04-2318-s018.pdf]

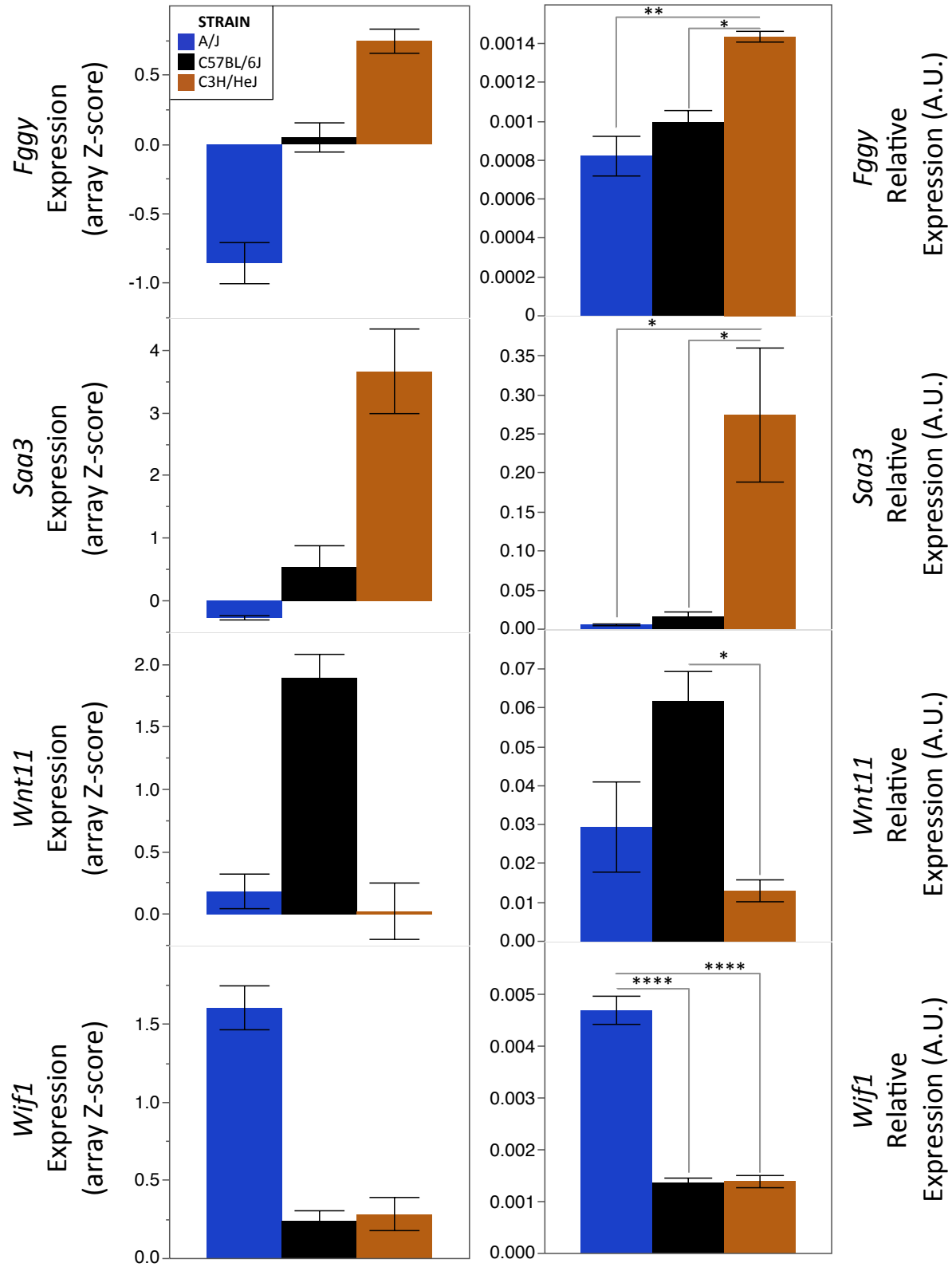

Supplement: Figure S11 — ΔCt = cycle threshold, normalized to the geometric mean of three control genes (Actb, Rpl10, Rpl13a). Variation between biological replicates (detected by microarray) did not significantly impact trends of strain-variation when quantified by qPCR. Each error bar constructed using one standard error from the mean. Significant differences detected by Tukey multiple comparisons ANOVA between strains. ∗P < 0.05, ∗∗P < 0.01, ∗∗∗∗P < 0.0001. [file peerj-04-2318-s020.pdf]
