# Supplementary material for: Temporal dynamics of the developing lung transcriptome in three common inbred strains of laboratory mice reveals multiple stages of postnatal alveolar development
Source: PeerJ. 2016 Aug 9;4:e2318. doi: 10.7717/peerj.2318 (PMC4991849; doi:10.7717/peerj.2318)
Supplement: Figure S6 — Heatmap showing the relative expression of 35 genes previously associated with cellular differentiation in the lung. Each expression profile is relative to the average expression of that gene across all time points. Solid blue indicates 1.5-fold decrease relative to average; yellow indicates 1.5-fold increase. Gene loading values (PC1-3) are shown to right of heatmap; dark red shading indicates that a gene is within the top 5% of contributors to that respective PC; light red or dark red squares indicate genes that were captured by the mDLCS. [file peerj-04-2318-s015.pdf]

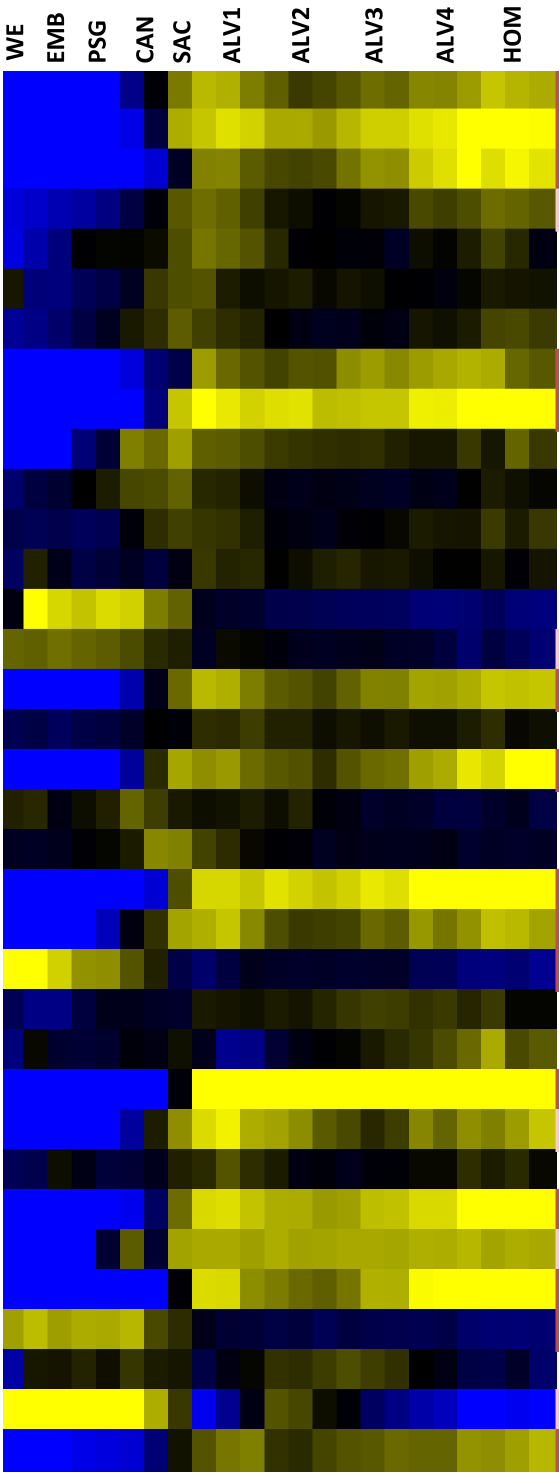

MGI ID	Symbol	PC1	PC2	PC3
MGI:1351617	*Abca3*	-0.96	0.16	0.10
MGI:893592	*Ager*	-0.97	0.17	0.06
MGI:106215	*Aqp5*	-0.97	-0.10	-0.02
MGI:99480	*Cebpa*	-0.89	0.13	0.26
MGI:88388	*Cftr*	-0.54	0.39	0.19
MGI:1306784	*Cited2*	-0.57	0.17	0.32
MGI:2447167	*Cmtm8*	-0.65	0.28	0.43
MGI:95537	*Ctgf*	-0.94	0.02	-0.07
MGI:1339941	*Cxcl15*	-0.96	0.16	0.06
MGI:1858599	*Egfl6*	-0.71	0.53	0.22
MGI:95523	*Fgfr2*	-0.22	0.51	0.47
MGI:1347474	*Foxj1*	-0.70	0.17	0.41
MGI:1914004	*Foxp1*	-0.51	-0.03	-0.30
MGI:2148705	*Foxp2*	0.82	0.25	0.13
MGI:1888513	*Fzd2*	0.88	0.30	-0.02
MGI:1891250	*Gprc5a*	-0.97	0.16	0.09
MGI:104853	*Hes1*	-0.70	0.14	0.01
MGI:1916782	*Hopx*	-0.94	0.11	0.22
MGI:96397	*Id2*	0.47	0.36	0.22
MGI:96689	*Krt15*	0.04	0.57	0.51
MGI:96897	*Lyz2*	-0.98	0.10	0.02
MGI:97231	*Muc1*	-0.91	0.28	0.18
MGI:97357	*Mycn*	0.95	-0.09	-0.16
MGI:97363	*Notch1*	-0.75	0.15	-0.31
MGI:97530	*Pdgfra*	-0.47	-0.28	0.08
MGI:98919	*Scgb1a1*	-0.96	-0.01	-0.07
MGI:2153470	*Scgb3a2*	-0.89	0.18	0.21
MGI:107558	*Sema3a*	-0.51	0.14	0.17
MGI:109516	*Sftpb*	-0.97	0.12	0.05
MGI:109517	*Sftpc*	-0.85	0.39	0.07
MGI:109515	*Sftpd*	-0.96	-0.08	0.03
MGI:98371	*Sox9*	0.93	0.12	0.11
MGI:98726	*Tgfb2*	0.07	0.69	-0.42
MGI:98790	*Top2a*	0.91	0.28	-0.12
MGI:103178	*Vegfa*	-0.97	-0.03	-0.05
