# Supplementary material for: Temporal dynamics of the developing lung transcriptome in three common inbred strains of laboratory mice reveals multiple stages of postnatal alveolar development
Source: PeerJ. 2016 Aug 9;4:e2318. doi: 10.7717/peerj.2318 (PMC4991849; doi:10.7717/peerj.2318)

A Wnt/Frizzled-related genes

PC1	PC2	PC3	PC4	PC5	PC6	PC7	PC8	PC9	PC10	strain	strain*stage	MGI ID	Symbol
-0.14	0.66	-0.52	0.08	-0.22	0.00	0.17	-0.08	-0.10	0.08	0.0614	0.0186*	MGI:1196625	Fzd1
0.73	-0.16	0.16	0.11	-0.05	-0.25	-0.43	0.04	0.12	-0.14	0.1248	<.0001**	MGI:2136761	Fzd10
0.88	0.30	-0.02	0.11	0.05	0.04	-0.07	-0.14	-0.08	0.04	0.0975	0.0039*	MGI:1888513	Fzd2
0.88	-0.21	-0.11	0.18	0.07	-0.18	-0.16	0.05	0.00	-0.01	0.0011*	<.0001**	MGI:108476	Fzd3
-0.91	0.15	-0.10	0.12	0.05	0.02	-0.06	0.13	0.01	-0.02	<.0001**	0.0080*	MGI:108520	Fzd4
-0.92	0.20	-0.07	0.09	0.05	-0.01	0.01	0.06	0.15	-0.06	<.0001**	0.0256*	MGI:108474	Fzd6
0.92	0.19	-0.05	0.11	-0.11	0.04	-0.03	-0.19	0.00	0.01	0.2761	0.0112*	MGI:1926790	Fzr1
0.87	-0.07	-0.04	0.02	-0.09	-0.06	-0.06	-0.08	-0.17	0.22	0.3767	<.0001**	MGI:108078	Sfrp2
-0.58	0.43	-0.40	-0.05	-0.07	-0.26	-0.02	0.04	0.28	-0.16	<.0001**	<.0001**	MGI:1344332	Wif1
-0.08	0.32	-0.72	0.09	-0.27	0.00	-0.08	-0.06	-0.02	0.09	0.3774	<.0001**	MGI:101948	Wnt11
0.44	0.66	0.01	-0.04	-0.06	0.18	0.23	-0.14	0.01	0.08	0.0213*	0.0004*	MGI:98954	Wnt2
0.64	-0.23	0.01	-0.07	-0.07	0.29	0.10	-0.27	-0.30	0.14	<.0001**	<.0001**	MGI:1261834	Wnt2b
-0.23	0.51	-0.49	0.06	0.03	-0.13	-0.39	0.33	0.00	-0.07	<.0001**	0.0027*	MGI:98958	Wnt5a
-0.76	-0.08	0.05	0.27	0.04	-0.06	-0.23	0.03	0.14	-0.04	0.9052	<.0001**	MGI:98961	Wnt7a
0.84	0.16	-0.01	-0.04	-0.07	0.23	0.22	-0.25	-0.08	0.10	0.038*	0.0444*	MGI:98962	Wnt7b

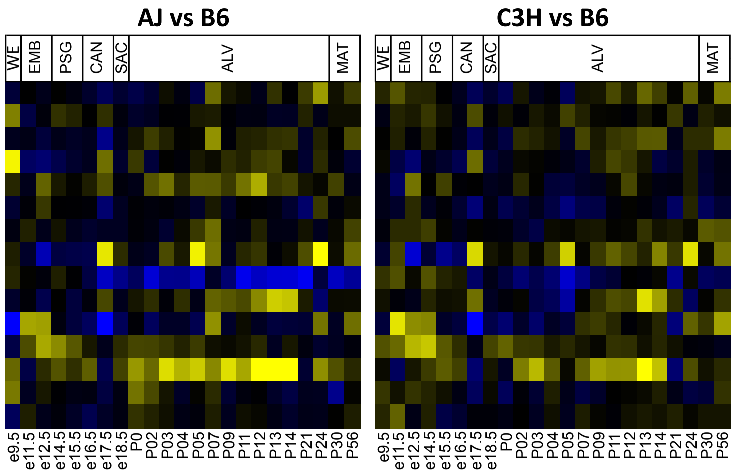

B Annotated with pulmonary fibrosis (MP:0006050)

PC1	PC2	PC3	PC4	PC5	PC6	PC7	PC8	PC9	PC10	strain	strain*stage	MGI ID	Symbol
-0.89	0.34	0.15	-0.06	0.03	0.12	0.18	0.00	0.03	-0.01	0.0346	0.2320	MGI:102709	Cav1
-0.66	-0.11	-0.05	0.30	0.12	-0.15	0.17	0.04	0.36	0.14	<.0001**	<.0001**	MGI:109177	Cd59a
-0.54	0.39	0.19	0.01	0.45	0.06	0.33	-0.09	0.16	0.03	<.0001**	<.0001**	MGI:88388	Cftr
-0.85	0.22	0.11	-0.06	0.13	-0.02	-0.20	-0.04	-0.06	0.17	0.1330	0.0695	MGI:103159	Cish
-0.92	0.15	-0.11	0.06	-0.15	0.05	-0.07	0.08	0.04	-0.01	0.0015	0.0089	MGI:2135755	Cox4i2
0.03	-0.01	0.18	-0.09	0.00	0.24	-0.25	0.09	0.03	0.04	0.0001	0.0002	MGI:1891837	Cpb2
-0.75	-0.24	-0.21	0.24	-0.07	0.03	0.15	0.14	0.21	0.21	<.0001**	<.0001**	MGI:107823	Ctsk
0.97	-0.05	0.02	0.15	0.02	-0.02	0.09	0.03	-0.01	-0.02	0.8643	0.4995	MGI:1861727	Dkc1
-0.56	0.00	-0.32	0.10	-0.08	0.11	-0.16	-0.13	0.05	0.11	0.0470	0.9061	MGI:95295	Egr1
-0.84	0.35	0.03	0.03	0.05	0.02	0.17	0.15	-0.01	-0.05	<.0001**	0.0012	MGI:102805	Entpd1
-0.73	-0.21	-0.12	0.26	0.11	0.02	0.15	0.06	0.24	0.23	<.0001**	<.0001**	MGI:96070	Hbegf
0.28	-0.24	-0.16	0.32	0.30	-0.10	0.16	0.19	0.40	0.43	0.8926	<.0001**	MGI:106911	Hccs
-0.82	-0.03	-0.08	0.16	-0.16	0.20	-0.20	0.21	-0.14	0.16	<.0001**	<.0001**	MGI:96052	Hck
-0.54	0.16	0.14	0.13	0.09	-0.07	-0.39	0.15	-0.04	-0.05	0.0376	0.0070	MGI:96396	Id1
-0.81	-0.13	0.04	0.21	0.22	-0.14	0.10	-0.01	0.06	0.14	0.0032	<.0001**	MGI:96820	Lpl
-0.75	0.31	0.06	0.05	0.45	0.00	-0.03	0.06	-0.01	0.07	0.0005	<.0001**	MGI:107448	Lyst
-0.81	0.33	0.36	0.06	0.04	0.05	0.13	-0.01	-0.07	-0.08	0.0026	0.4401	MGI:108420	Nfe2l2
-0.29	-0.21	-0.55	0.19	-0.15	-0.10	-0.08	0.02	-0.21	0.26	<.0001**	0.0004	MGI:97610	Plat
0.00	0.02	0.17	-0.13	-0.02	0.19	-0.30	0.03	0.02	0.05	0.0001	0.0005	MGI:97620	Plg
-0.87	-0.08	0.24	0.14	0.07	0.03	0.13	0.03	0.19	0.13	<.0001**	<.0001**	MGI:97747	Pparg
-0.89	-0.18	0.15	0.06	0.04	0.01	0.08	-0.03	-0.05	0.18	0.0140	0.0246	MGI:109284	Psen2
-0.77	-0.42	0.15	0.18	-0.07	-0.03	0.13	-0.16	-0.03	-0.02	<.0001**	<.0001**	MGI:97798	Ptgs2
-0.96	-0.08	0.03	0.05	-0.01	-0.04	-0.11	0.05	-0.08	0.08	0.0001	<.0001**	MGI:109515	Sftpd
-0.63	-0.33	0.11	0.21	-0.03	0.08	0.09	0.14	0.13	0.22	<.0001**	0.1768	MGI:98389	Spp1
-0.34	0.02	-0.20	0.05	0.26	-0.38	-0.03	-0.09	0.35	-0.12	<.0001**	<.0001**	MGI:98385	Spta1
-0.85	0.41	0.05	0.06	-0.09	0.14	0.07	-0.14	-0.04	0.07	0.0011	0.0537	MGI:1314884	Tnfrsf1a
-0.93	-0.10	-0.16	0.17	-0.06	-0.04	0.00	0.05	-0.01	0.04	0.0084	0.9569	MGI:1314883	Tnfrsf1b
0.93	0.03	-0.20	0.12	-0.12	0.01	0.04	-0.04	0.01	0.05	0.1260	0.6280	MGI:98834	Trp53
-0.93	0.26	0.01	0.04	0.08	0.01	0.01	-0.08	0.06	-0.09	<.0001**	0.0001	MGI:1917649	Wwtr1

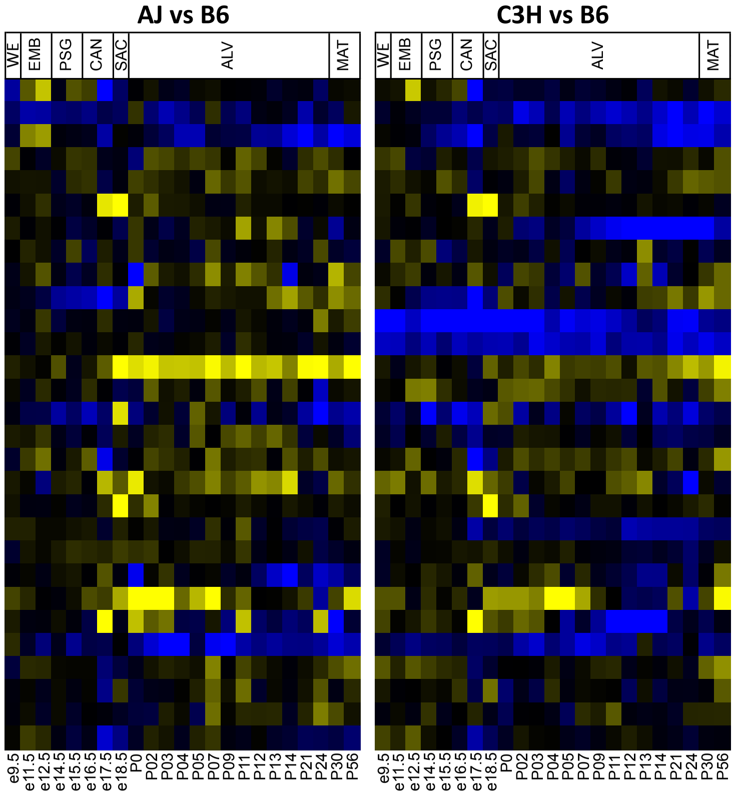

Supplement: Figure S10 — Light red highlighting indicates genes that were included in the mDLCS; dark red highlighting indicates genes in the top 5% of contributors to a given PC. Heatmaps contrast expression levels in A/J (left) or C3H/HeJ (right) with C57BL/6J. Solid blue indicates a 1-fold increase in expression between strains; solid yellow indicates a 1-fold decrease in expression between strains. [file peerj-04-2318-s019.pdf]
