# Supplementary material for: Temporal dynamics of the developing lung transcriptome in three common inbred strains of laboratory mice reveals multiple stages of postnatal alveolar development
Source: PeerJ. 2016 Aug 9;4:e2318. doi: 10.7717/peerj.2318 (PMC4991849; doi:10.7717/peerj.2318)
Supplement: Table S1 — PCA-derived loading values (for PC1-3) also shown for each of these genes. [file peerj-04-2318-s021.docx]

| **Gene Symbol** | **MGI ID** | **PC1** | **PC2** | **PC3** |
| --- | --- | --- | --- | --- |
| **Toll-like receptors** | | | | |
| *Tlr2*  *Tlr3*  *Tlr4*  *Tlr5*  *Tlr7*  *Tlr8*  *Tlr13* | MGI:1346060  MGI:2156367  MGI:96824  MGI:1858171  MGI:2176882  MGI:2176887  MGI:3045213 | -0.82  -0.93  -0.93  -0.89  -0.80  -0.85  -0.82 | -0.25  -0.06  -0.17  -0.06  -0.30  -0.22  0.00 | 0.21  0.02  0.08  -0.10  0.02  0.08  0.07 |
| **Lymphocyte antigen complexes** | | | | |
| *Ly6a*  *Ly6c1*  *Ly6e*  *Ly86* | MGI:107527  MGI:96882  MGI:106651  MGI:1321404 | -0.95  -0.96  -0.94  -0.84 | -0.09  -0.10  -0.02  0.06 | -0.08  -0.02  -0.08  0.04 |
| **Chemokine receptors/ligands** | | | | |
| *Ccl6*  *Ccr2*  *Ccrl2*  *Cxcl15*  *Cxcl16*  *Cxcr4* | MGI:98263  MGI:106185  MGI:1920904  MGI:1339941  MGI:1932682  MGI:109563 | -0.97  -0.96  -0.90  -0.89  -0.82  -0.78 | -0.97  -0.96  -0.90  -0.89  -0.82  -0.78 | -0.08  0.06  -0.16  -0.03  0.05  -0.21 |
| **Interleukins** | | | | |
| *Ighm*  *Igkc*  *Ifkj1*  *Igsf5*  *Igsf6*  *Igsf9*  *Ikbkap*  *Il10rb*  *Il16*  *Il17ra*  *Il17rc*  *Il17re*  *Il18r1*  *Il1r1*  *Il1rl2*  *Il2rg*  *Il33*  *Il34*  *Il4ra*  *Il6ra*  *Il6st*  *Ildr1*  *Ilf2*  *Ilf3* | MGI:96448  MGI:96495  MGI:1316689  MGI:1919308  MGI:1891393  MGI:2135283  MGI:1914544  MGI:109380  MGI:1270855  MGI:107399  MGI:2159336  MGI:1889371  MGI:105383  MGI:96545  MGI:1913107  MGI:96551  MGI:1924375  MGI:1923777  MGI:105367  MGI:105304  MGI:96560  MGI:2146574  MGI:1915031  MGI:1339973 | -0.81  -0.84  -0.81  -0.87  -0.80  0.93  0.95  -0.93  -0.89  -0.89  -0.90  -0.82  -0.95  -0.92  -0.77  -0.95  -0.92  -0.86  -0.97  -0.89  -0.93  -0.85  0.98  0.97 | -0.24  -0.30  -0.35  -0.04  0.08  -0.28  -0.14  0.14  0.08  0.00  0.11  -0.13  -0.04  0.16  0.42  0.05  -0.11  -0.13  -0.01  -0.01  -0.02  -0.16  0.04  0.07 | -0.30  -0.19  -0.18  0.33  0.05  -0.07  -0.14  0.25  0.16  -0.08  0.12  0.17  0.15  0.10  -0.12  -0.08  0.02  0.33  -0.08  0.23  0.04  0.19  0.02  -0.13 |
