# Supplementary material for: Temporal dynamics of the developing lung transcriptome in three common inbred strains of laboratory mice reveals multiple stages of postnatal alveolar development
Source: PeerJ. 2016 Aug 9;4:e2318. doi: 10.7717/peerj.2318 (PMC4991849; doi:10.7717/peerj.2318)
Supplement: Table S2 — Mean differences (Mean diff.) in relative expression units (A.U.) between strains analyzed by Tukey multiple testing corrected ANOVA. Results predominately conform to the trends of strain-dependent expression detected by microarray (shown next to each gene name on left) as seen by comparing qPCR significance to regression significance. [file peerj-04-2318-s022.pdf]

Tukey's multiple comparisons test		Mean diff.	95% confidence limits	Adjusted p value	Regression significant?	qPCR significant?
***Fggy* (AJ < B6 < C3H)**						
A/J vs. C57BL6J	A-B	-0.0001722	-4.798x10^{-4} to 1.353x10^{-4}	0.2738	Yes	No
A/J vs. C3H/HeJ	A-C	-0.0006147	-9.222x10^{-4} to -3.071x10^{-4}	0.0021	Yes	Yes
C57BL6J vs. C3H/HeJ	B-C	-0.0004424	-7.499x10^{-4} to -1.349x10^{-4}	0.0107	Yes	Yes
***Saa3* (AJ < B6 << C3H)**						
A/J vs. C57BL6J	A-B	-0.01083	-0.22650 to 0.20480	0.9870	No	No
A/J vs. C3H/HeJ	A-C	-0.2689	-0.48450 to -0.05319	0.0204	Yes	Yes
C57BL6J vs. C3H/HeJ	B-C	-0.258	-0.47370 to -0.04236	0.0243	Yes	Yes
***Wnt11* (AJ << B6 >> C3H)**						
A/J vs. C57BL6J	A-B	-0.03237	-0.06789 to 0.00316	0.0702	Yes	No
A/J vs. C3H/HeJ	A-C	0.01641	-0.01911 to 0.05193	0.3909	No	No
C57BL6J vs. C3H/HeJ	B-C	0.04878	0.01326 to 0.08430	0.0132	Yes	Yes
***Wif1* (AJ >> B6 ≈ C3H)**						
A/J vs. C57BL6J	A-B	0.003328	0.002553 to 0.004103	<0.0001	Yes	Yes
A/J vs. C3H/HeJ	A-C	0.003306	0.002531 to 0.004081	<0.0001	Yes	Yes
C57BL6J vs. C3H/HeJ	B-C	-2.152e-05	-7.96x10^{-4} to 7.54x10^{-4}	0.9960	No	No
